# Delineating inflammatory and mechanical sub-types of low back pain: a pilot survey of fifty low back pain patients in a chiropractic setting

**DOI:** 10.1186/2045-709X-19-5

**Published:** 2011-02-07

**Authors:** Janine S Riksman, Owen D Williamson, Bruce F Walker

**Affiliations:** 1 School of Chiropractic and Sports Science, Murdoch University, South Street, Murdoch WA, 6151, Australia; 2 Department of Epidemiology and Preventive Medicine Monash University, The Alfred Centre, 99 Commercial Road, Melbourne VIC, 3004, Australia

## Abstract

**Background:**

An instrument known as the Mechanical and Inflammatory Low Back Pain (MAIL) Scale was drafted using the results of a previous expert opinion study. A pilot survey was conducted to test the feasibility of a larger study designed to determine the MAIL Scale's ability to distinguish two potential subgroups of low back pain: inflammatory and mechanical.

**Methods:**

Patients with a primary complaint of low back pain (LBP) presenting to chiropractic clinics in Perth, Western Australia were asked to fill out the MAIL Scale questionnaire. The instrument's ability to separate patients into inflammatory and mechanical subgroups of LBP was examined using the mean score of each notional subgroup as an arbitrary cut-off point.

**Results:**

Data were collected from 50 patients. The MAIL Scale did not appear to separate cases of LBP into the two notionally distinct groups of inflammatory (n = 6) or mechanical (n = 5). A larger "mixed symptom" group (n = 39) was revealed.

**Conclusions:**

In this pilot study the MAIL Scale was unable to clearly discriminate between what is thought to be mechanical and inflammatory LBP in 50 cases seen in a chiropractic setting. However, the small sample size means any conclusions must be viewed with caution. Further research within a larger study population may be warranted and feasible.

## Background

Low back pain (LBP) is a common condition, with about 79% of Australians experiencing LBP at some time in their lives [1]. In over 85% of cases presenting for primary care [2] a specific cause for pain cannot be identified [3]. In such cases, the LBP is often labelled as non-specific low back pain (NSLBP).

Over 90% of primary contact clinicians believe NSLBP is not a single condition, with three-quarters believing subgroups are already identifiable [4]. However there is little current evidence supporting the existence of these subgroups, or agreement between practitioners when defining their characteristics [5].

A recent study has shown that people diagnosed with NSLBP might be categorised as having mechanical (MLBP) or inflammatory (ILBP) low back pain [6]. In this study of expert opinion, a number of signs were identified as potentially indicating LBP of mechanical origin [6]. These were intermittent pain during the day, pain that develops later in the day, pain on standing for a while, pain with lifting, pain with bending forward a little, pain on trunk flexion or extension, pain on doing a sit up, pain when driving long distances, pain getting out of a chair and pain on repetitive bending, running, and coughing or sneezing.

Similarly, other studies suggest that ILBP might be defined by pain that wakes the person, pain on morning waking, pain associated with morning stiffness longer than 30 minutes and improvement of LBP with exercise but not rest [6-8].

Additionally, several studies into treatment-based classification have shown that people with certain clinical signs and symptoms may exhibit a preferential response to corresponding treatment modalities [9-12].

The aim of this study was to pilot a survey that tests the feasibility of discriminating cases of NSLBP into two subgroups, mechanical and inflammatory, based on clinical signs and symptoms.

## Methods

### Sample Population

This study included consecutive patients with a primary complaint of LBP who voluntarily presented for treatment to the Murdoch University Chiropractic Clinic and three private chiropractic practices in Perth, Western Australia, from March 2008 until July 2008. The study was approved by the Murdoch University Human Research Ethics Committee.

Any patient aged 18 years or older with a primary complaint of LBP, with or without referral to the lower extremity was eligible for inclusion. Exclusion criteria included those with the of presence any 'red flags' for serious spinal pathology (for example, tumour, fracture or infection), prior surgery to the lumbar spine, pregnancy, diagnosed bipolar disorder or schizophrenia, seeking legal advice regarding their condition or claiming treatment under a Worker's Compensation/Third Party insurance claim and finally chiropractic students. Chiropractic students were excluded as patient participants, as some had prior knowledge of the study methods.

LBP was defined as any pain in the region between the lower ribs and gluteal folds [13].

### Patient Assessment

All patients received routine questions on LBP history and a physical examination of active lumbar range of motion, lower limb neurological examination (reflexes, sensation and motor strength), straight leg raise and various orthopaedic tests professed to identify dysfunction in the lumbopelvic region. Levels of pain, disability and fear avoidance beliefs were recorded using a numerical rating scale (NRS), Oswestry Disability Questionnaire (ODQ) and Fear Avoidance Beliefs Questionnaire (FABQ), respectively.

### Measures of Health Status

Baseline demographics, self-reported pain and disability data were collected on all patients prior to treatment. The location of LBP was assessed using a body pain diagram. Current pain severity was assessed on an 11-point numerical rating scale (NRS), ranging from 0 (no pain) to 10 (worst pain imaginable) [14,15] and LBP-related disability was measured using an Oswestry Disability Questionnaire (ODQ) [16].

### The Mechanical and Inflammatory Low Back Pain Scale (MAIL Scale)

**Figure 1:**
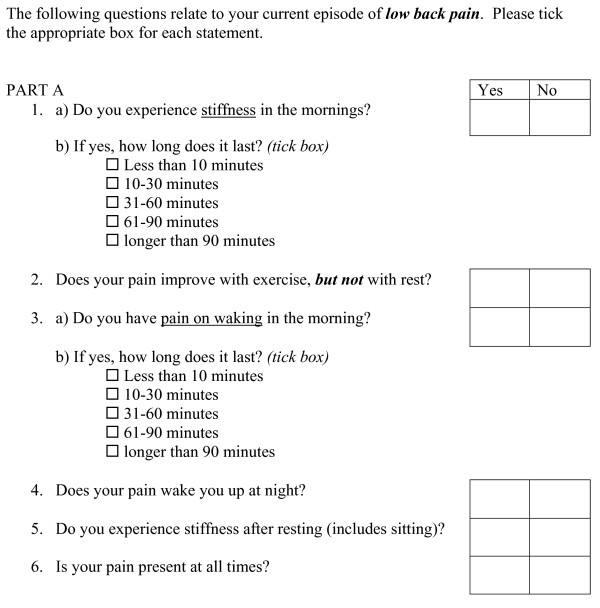
**Mechanical and Inflammatory Low Back (MAIL) Scale - Part A**.

**Figure 2:**
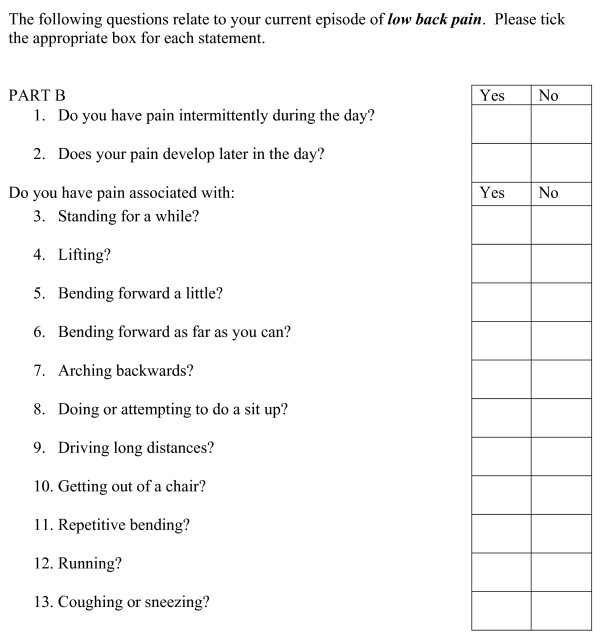
**Mechanical and Inflammatory Low Back (MAIL) Scale - Part B**. Scoring key: SCORE: Part A ____/16 = _______% Part B ____/13 = _______% Scoring ■ Questions 1 and 3 in Part A attract 2 points each for a 'yes response'. ■ All other questions in Parts A and B attract one point for a 'yes' response, zero points for a 'no' response. ■ The five categories of questions 1b and 3b are scored from 0 to 4 points, with zero points for duration of <10 minutes, progressing to 4 points for >90 minute category. ■ The maximum score possible in Part A is 16 points and Part B is 13 points.

Also at baseline, each study participant filled out the new instrument known as the Mechanical and Inflammatory Low Back Pain (MAIL) Scale, (Figures 1 and 2).

The MAIL Scale, comprised three parts and asked the patient to answer 'yes' or 'no' to a series of 19 questions relating to notional mechanical and inflammatory back pain.

Part A consists of 6 signs and symptoms thought to characterise inflammatory pain (Figure 1). Of these, questions 1 and 2 were derived from the clinical questionnaire administered by Rudwaleit et al [8], which showed morning stiffness and relief of pain with exercise (but not rest) to be independently associated with inflammatory back pain. Question 1b provides time frames to measure the duration of morning stiffness as Rudwaleit et al [8] found morning stiffness greater than 30 minutes distinguished inflammatory from mechanical pain. Questions 3 through 6 are those signs and symptoms thought to be associated with pain of a non-specific inflammatory nature by experts surveyed by Walker and Williamson [6]. Question 3b similarly provides time frames to measure the duration of morning pain. The potential answers were weighted in a subjective manner with weighting rising with increasing duration of morning pain or stiffness as this variable is thought to be strongly associated with ILBP [6,8].

Part B consists of 13 signs and symptoms thought to characterise MLBP (Figure 2) [6]. From the completed MAIL Scale, an arbitrary weighted score was generated for the number of mechanical and inflammatory characteristics exhibited by each patient. The spread of responses to the MAIL Scale was analysed in order to determine its preliminary ability to discriminate patients into categories.

### Sample Size

For this pilot study, an arbitrary sample size of 50 was used. Sample size calculations for a fully powered study may be derived from the pilot study results.

### Analysis

Data were entered, cleaned and analysed using Statistical Package for the Social Sciences (SPSS) version 16.0 [17]. Normality tests were performed on both MAIL Scale subgroups using a Shapiro-Wilk test in SPSS [17]. Descriptive statistics were used to analyse the scores and frequencies of responses to the MAIL Scale instrument questions. A Pearson's correlation test between the Inflammatory and Mechanical subscales was also performed.

Subject to normality being shown, the mean scores of each of the Inflammatory (Part A) and Mechanical (Part B) subscales of the MAIL Scale would be used as a notional and arbitrary cut-off point to indicate "Inflammatory LBP" and "Mechanical LBP". Those with scores greater than the mean in one subscale and less than the mean in the other subscale were categorised as either purely inflammatory or mechanical LBP. Any MAIL Scale scores that did not meet these criteria were categorised as "mixed" LBP.

## Results

### Patient Characteristics

Data were collected from all 50 patients in the pilot study; their main clinical features are presented in Table 1.

**Table 1:** Baseline data and subject characteristics

	Mean (SD)
**Age (years)**	37.0 (15.5)

**Gender**	Male = 29 (58.0%)

NRS* *(0-10)*	5.3 (2.0)

**ODQ**^†^	22.5% (16.1%)

**MAIL Scale**^‡^	6.9 (4.0)
**- Inflammatory ***(0-16)*	

**MAIL Scale**^‡^	7.7 (3.2)
**- Mechanical ***(0-13)*	

**FABQ**^§^	12.2 (5.2)
**- Physical Activity ***(0-30)*	

* Numerical Rating Scale

^† ^Oswestry Disability Questionnaire

^‡ ^Mechanical and Inflammatory Low Back Pain Scale

^§ ^Fear Avoidance Belief Questionnaire

Of the 50 included patients, 38 were recruited through Murdoch University Chiropractic Clinic and 12 through private chiropractic practices in the Perth metropolitan area.

### Mechanical and Inflammatory Low Back Pain Scale (MAIL Scale)

The MAIL Scale scores for all participants are shown in Table 1, with a mean inflammatory score of 6.9 (43.1%) out of a possible 16 and mean mechanical score of 7.7 (59.2%) out of a possible 13. Data were tested for normality using a Shapiro-Wilk calculation which showed the data for both subgroups were normally distributed (S-W 0.98, df 50, p = 0.5). An additional file containing MAIL Scale raw data for individual items is attached (See additional files 1 and 2: MAIL Scale Raw Scores and Mail Scale Variable Legend).

The number of positive and negative responses to each of the inflammatory and mechanical signs and symptoms are listed in Table 2.

**Table 2:** Frequency of MAIL Scale responses

Signs and symptoms	No	Yes	*n *responding
**PART A - Inflammatory**			

Morning stiffness	10 (20%)	40 (80%)	50

Duration of Morning stiffness			49
Did not have to answer	10 (20.0%)		
<10 mins	10 (20.0%)		
10-30 mins	18 (36.7%)		
31-60 mins	5 (10.2%)		
61-90 mins	0 (0.0%)		
>90 mins	6 (12.2%)		

Improvement of pain with exercise, but not rest	22 (44.9%)	27 (55.1%)	49

Morning pain on waking	19 (38.8%)	30 (61.2%)	49

Duration of morning pain			45
Did not have to answer	19 (42.2%)		
<10 mins	7 (15.6%)		
10-30 mins	10 (22.2%)		
31-60 mins	2 (4.4%)		
61-90 mins	0 (0.0%)		
>90 mins	7 (15.6%)		

Pain that wakes	31 (62%)	19 (38%)	50

Stiffness after resting (includes sitting)	4 (8%)	46 (92%)	50

Pain present at all times	30 (60%)	20 (40%)	50

**PART B - Mechanical**			

Intermittent pain during day	18 (36.7%)	31 (63.3%)	49

Pain developing later in the day	33 (68.8%)	15 (31.2%)	48

Pain with standing for a while	15 (30.6%)	34 (69.4%)	49

Pain with lifting	14 (29.2%)	34 (70.8%)	48

Pain with bending forward a little	16 (32.7%)	33 (67.3%)	49

Pain on bending forward as far as you can	19 (38%)	31 (62%)	50

Pain on arching backwards	15 (30%)	35 (70%)	50

Pain on doing or attempting a sit-up	16 (32%)	34 (68%)	50

Pain on driving long distances	18 (36%)	32 (64%)	50

Pain on getting out of a chair	23 (46%)	27 (54%)	50

Pain on repetitive bending	13 (26%)	37 (74%)	50

Pain on running	22 (44%)	28 (56%)	50

Pain on coughing or sneezing	34 (70.8%)	14 (29.2%)	48

'Morning stiffness' and 'Stiffness after resting' received the most positive responses in the inflammatory section, with 80% (n = 40) and 92% (n = 46) of patients answering "yes", respectively. In the mechanical section 'Pain on repetitive bending' (74%, n = 37), 'Pain on lifting' (70.8%, n = 34) and 'Pain on arching backwards' (70%, n = 35) were the most prevalent.

Seven patients (14%) responded "yes" and 1 (2%) responded "no" to all six inflammatory signs and symptoms. The number of patients responding "yes" to all 13 mechanical signs and symptoms was 2 (4%), with 1 (2%) patient responding "no" to all. There were no reports of participants having difficulty completing the MAIL Scale.

**Figure 3:**
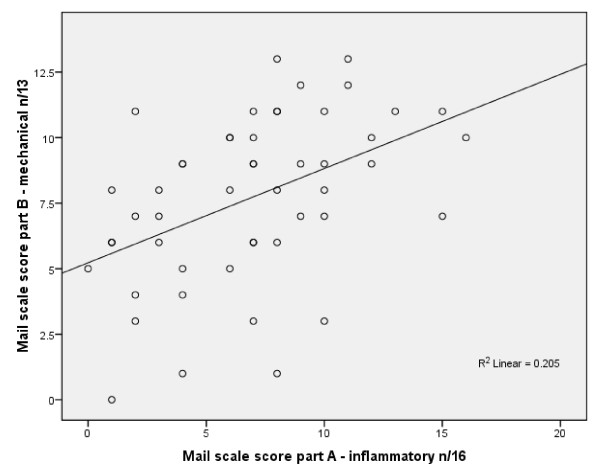
**Scatterplot showing correlation between MAIL Scale Part A Inflammatory and Part B Mechanical scores**.

The Pearson correlation co-efficient assessing the general relationship between the MAIL Scale Part A Inflammatory and Part B Mechanical scores was calculated and showed a positive correlation of r = 0.45, p = 0.01. This is shown as a scatterplot in Figure 3.

**Figure 4:**
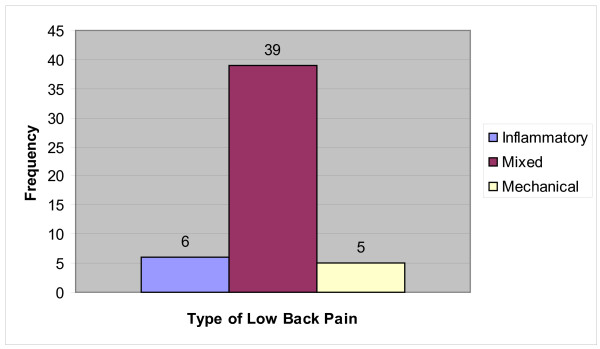
**Frequency of "Inflammatory LBP" (MAILS A >7 and MAILS B < 8), "Mechanical LBP" (MAILS A < 7 and MAILS B > 8) and "Mixed" type of Low Back Pain**.

The mean scores of the Inflammatory and Mechanical subscales of the MAIL Scale were used as an arbitrary cut-off point to classify "Inflammatory LBP" and "Mechanical LBP". Those with scores greater than the mean in one subscale and less than the mean in the other subscale were categorised as either purely inflammatory or mechanical LBP. By this method, 6 cases were classified as ILBP, 5 cases were classified as MLBP, with the remaining 39 cases classified as "mixed LBP". The frequency of each 'type' of LBP is shown in Figure 4.

## Discussion

### Introduction

This study found that the MAIL Scale was easy and relatively quick for participants to complete, but was unable to effectively categorise the majority of patients into either inflammatory or mechanical LBP. There are many reasons for this including that the sample size was too small to detect a difference (Type II error), the instrument is unable to distinguish between these two notional categories, the concept of mechanical and inflammatory causes in NSLBP is not valid, the population of patients did not have severe enough forms of NSLBP to be detected by the instrument or the majority of patients have mixed patterns of NSLBP. Of those 50 who entered the study, characteristics for age, sex and measures of health status (Table 1) are similar to those recorded in prior studies conducted in chiropractic teaching facilities [18].

### Mechanical and Inflammatory Low Back Pain (MAIL) Scale

The items in this questionnaire were selected based on expert opinion and a search of current literature, and thereby have a level of both face and content validity [19].

While the MAIL Scale was unable to discriminate LBP into two groups, certain signs and symptoms appeared more prominently in each subscale and may be important for future research into this area.

Part A of the MAIL Scale dealt with those signs and symptoms thought to be associated with inflammatory LBP (Table 2). 'Stiffness after resting (includes sitting)' scored the highest "yes" response (92%) in this section. This sign is commonly regarded as an inflammatory symptom in a rheumatological context as the inflammatory mediators, cytokines, are strongly involved in the synovial immune and inflammatory response in conditions such as rheumatoid arthritis [20]. The presence of these cytokines may result in a "gelling" phenomenon, whereby a period of inactivity results in an accumulation of inflammatory mediators in the involved area. As the person then gets up to move, stiffness is experienced in the area until there has been sufficient movement to disperse the accumulated inflammation [20].

It is worthy of note that stiffness after seated rest may also have a mechanical cause as it has also been attributed to intervertebral disc herniation of the lumbar spine. The discs at the L4-5 and L5-S1 levels bear high loads [21,22] and in the seated position intradiscal pressure has shown increases to 100 kilograms of force (kgf), from 70 kgf in the standing position [23]. Therefore in a patient with a suspected lumbar spine disc lesion such as herniation, stiffness after resting (particularly in the seated position) may be a poor discriminating symptom of "mechanical" or "inflammatory" back pain in the absence of further clinical information.

'Morning stiffness' scored the second highest "yes" response (40 patients, 80%) and of those 40 patients, 28 (56.7%) experienced stiffness for 30 minutes or less, while for 11 (22.4%), the stiffness lasted over 30 minutes. Six of these 11 participants also experienced morning pain for longer than 30 minutes. In previous studies of morning stiffness, durations of greater than 30 minutes seemed to be the agreed threshold for determining the presence of inflammation [7,8,24]. As such, while a large number of LBP patients report experiencing morning stiffness in our study, less than a quarter were possibly attributable to an inflammatory aetiology based on the arbitrary scoring system used. It may be that systemic inflammation related to infection, or spondylo-arthropathies are more likely to be associated with these symptoms and not those of similar symptoms in NSLBP. The inflammatory back pain criteria developed by Rudwaleit [12] were centred on back pain as a result systemic inflammation associated with ankylosing spondylitis. The use of these criteria in the development of the MAIL Scale makes the assumption that non-systemic inflammation would give similar but localised symptoms. However, this may not be the case. An alternate study design using blood inflammatory markers (i.e. ERS, CRP) as an external reference standard may assist in the detection of existing inflammation. However, while these markers will detect systemic inflammation, a study into chronic LBP has shown that significant systemic inflammatory reaction was absent in the 273 participants sampled [25]. As such, while symptoms of apparent inflammation may be reported by these patients, objective signs may still yield sub-threshold measurements.

Part B of the MAIL Scale dealt with MLBP (Table 2). The relatively high proportion of patients responding "yes" to these "mechanical questions" is not unexpected when the biomechanics of these activities is considered. As mentioned previously, different activities result in varied loads and mechanical stresses to the spine. It is known that bending in various directions increases load on the elements of lumbar spine [23]. Repetition of this action may cause hysteresis [21] implying that the body is less protected against repetitive loads. This may also partly explain the high "yes" response rate to 'pain on driving long distances' due to the repetitive axial vibration in combination with the prolonged loading associated with sitting.

When lumbar flexion is coupled with lifting, the load increases significantly. Lifting a 20 kg weight with a flexed spine and straight knees results in 340 kgf load on the lumbar spine [23]. With proper lifting technique, limiting spinal flexion and 'lifting through' bent knees, the load on the spine is less (210 kgf), however this still represents a significant mechanical force on the spine. Similarly, activities such as performing a sit-up also increase the load on the spine, exerting 180 kgf to the lumbar discs.

'Pain developing later in the day' and 'Pain on coughing or sneezing' did not appear commonly, with only 15 (31.2%) and 14 (29.2%) of subjects responding "yes" respectively. The small number experiencing pain with coughing or sneezing may relate to the fact that only one respondent was diagnosed as having a disc herniation, and the presence of pain with these actions is commonly regarded as suggestive of a disc injury [26].

The correlation between the mechanical and inflammatory subscales of the MAIL Scale showed a significant positive correlation of 0.45 (Figure 3). This suggests that a distinction between mechanical and inflammatory LBP may not exist, and that the MAIL Scale is unable to separate LBP into two groups. In addition a negative correlation would have been expected if the LBP was caused predominantly by an inflammatory or mechanical cause.

LBP was arbitrarily classified into "inflammatory", "mechanical" or "mixed" subgroups. Over three-quarters of the sample were classified into the "mixed" subgroup, with only 6 and 5 patients classified as "inflammatory" and "mechanical" cases, respectively (Figure 4). This difficulty in discriminating notional mechanical from inflammatory pain is consistent with the original research [6], where experts were unable to clearly delineate those characteristics that were exclusive to either type of pain. However, the small sample size in this study may limit the conclusions that can be drawn. With a larger more diverse study population, separation into subgroups may or may not become more evident.

### Limitations and future research

The aim of this pilot study was to test the feasibility of a survey instrument that could potentially differentiate between the notional subgroups "inflammatory" and "mechanical" LBP. The results are not encouraging within the setting we chose.

We have shown that the frequency with which participants respond to each question in the MAIL Scale can be described, and their MAIL Scale scores derived. This can be used to assign them to an arbitrary subgroup classification, however it is less clear what contribution this makes.

The potential division of LBP into mechanical and inflammatory sub-groups based on an instrument of this type may rely on a much larger sample size. Analysis of a larger sample using an item-response theory approach such as Rasch analysis [27] would allow determination of which of these items are unidimensional, the hierarchy of those items and the most appropriate scoring system.

In any future research it may be best to recruit participants from other healthcare environments (such as clinics of physiotherapists, general practitioners and rheumatologists) as this may give a broader and more representative sample and decrease the potential for any selection bias. The broader spread of signs and symptoms may improve the ability of the instrument to discriminate between potential subgroups. In addition, consideration may be given to surveying active spondyloarthropathy patients from rheumatology clinics to ascertain whether they have a different frequency of the so-called mechanical signs and symptoms identified and present in the MAIL Scale instrument. It may be hypothesised that these patients have predominantly inflammatory pain and should exhibit less mechanical signs and symptoms that the NSLBP group.

Finally, it may be worth considering an alternative study design that uses an external reference standard, such as blood inflammatory markers which may assist the analysis by identifying systemic inflammatory cases. Depending on the study design selected, a sample size would need to be generated for a fully powered study. A power calculation has not been performed here, however the results shown in Table 1 may help with this calculation.

## Conclusion

In this pilot study, the MAIL Scale was simple to administer but was unable to clearly discriminate between notional mechanical and inflammatory LBP in a chiropractic setting. Sample size restrictions and the research setting limit any conclusions from these findings. Further research with a larger and more diverse study population may be warranted. However, based on the findings in this pilot study, separation of NSLBP into mechanical and inflammatory subgroups may not be possible.

## Competing interests

BW is the Editor in Chief of the journal Chiropractic & Osteopathy.

JR and OW declare that they have no competing interests.

## Authors' contributions

JR contributed to the design, carried out the data collection, performed the literature search, and drafted and wrote the manuscript which is based on her Honours thesis. BW and OW contributed to the supervision, concept and design, and editing and revision for the intellectual content of the article. BW provided statistical advice, and critical review of the manuscript. All authors read and approved the final manuscript.

## Supplementary Material

Additional file 1 **Mail Scale Raw Scores**. An additional file containing the raw data of 50 subjects for individual items on the MAIL Scale. Click here for file

Additional file 2 **Mail Scale Variable Legend**. An additional file containing the variable view of an SPSS data output and explanations of any abbreviations/numerical legends used within the MAIL Scale raw scores spreadsheet (additional file 1). Click here for file
